# Service quality in hospital inpatient care: SERVQUAL model approach

**DOI:** 10.4102/hsag.v30i0.3055

**Published:** 2025-09-30

**Authors:** Sitti Rahmatia, Muhammad Basri, Ismail Ismail, Sukriyadi Adi, Nasrullah Nasrullah, Aini Ahmad

**Affiliations:** 1 Department of Nursing, Makassar Health Polytechnic, South Sulawesi, Indonesia; 2 Faculty of Nursing, KPJ Health Care University, Kuala Lumpur, Malaysia

**Keywords:** service quality, inpatient care, SERVQUAL, patient satisfaction, hospital management

## Abstract

**Background:**

The quality of inpatient health care services significantly influences patient satisfaction, shaping perceptions of hospital performance and health care accessibility.

**Aim:**

This study examines patient satisfaction with inpatient services at Batara Siang Hospital, Indonesia, using the SERVQUAL model.

**Setting:**

The study was conducted in the inpatient wards of Batara Siang Hospital, a public health care facility in Pangkep, Indonesia.

**Methods:**

A cross-sectional observational study was conducted among 267 inpatients aged 25–50 years. Data were collected using a SERVQUAL-based questionnaire assessing five service quality dimensions, namely tangibility, reliability, responsiveness, assurance and empathy. Descriptive and multiple linear regression analyses were performed to determine the influence of each SERVQUAL dimension on overall patient satisfaction. Ethical approval was obtained from the Health Research Ethics Committee of Makassar Health Polytechnic.

**Results:**

The study found that all five SERVQUAL dimensions significantly influenced patient satisfaction. Empathy had the strongest impact (β = 0.629, *p* < 0.001), followed by assurance (β = 0.502, *p* < 0.001), responsiveness (β = 0.479, *p* < 0.001), reliability (β = 0.315, *p* = 0.01) and tangibility (β = 0.305, *p* = 0.04). These dimensions collectively explained 67.7% of the variance in patient satisfaction, indicating the effectiveness of the SERVQUAL model in evaluating hospital service quality.

**Conclusion:**

Enhancing empathy, assurance and responsiveness can significantly improve patient satisfaction. Hospitals should prioritise patient-centred communication, staff competency and responsiveness to optimise health care experiences and trust.

**Contribution:**

This study provides valuable insights for health care administrators and policymakers, offering practical strategies to enhance inpatient service quality.

## Introduction

Health care service quality has become an essential component of modern health care systems, influencing patient satisfaction, trust and overall health outcomes (Limbong, Sutinah & Agustina 2024). The quality of inpatient services is particularly critical as hospital environments directly impact patient experiences and perceptions of care (Bhaladhare & Rishipathak 2024). Ensuring high-quality inpatient services requires a comprehensive evaluation of various service dimensions, including the physical environment, provider competence, communication effectiveness and responsiveness to patient needs (Liu et al. 2024; Sihombing, Nasution & Zulfendri 2024). The increasing expectations of patients, driven by greater awareness of health care rights and service standards, have heightened the need for systematic approaches to evaluating and improving health care service quality (Ferreira et al. 2023; Ghali, Garrouch & Aljasser 2023; Tarafder 2024). Various models have been developed to measure service quality, among which the SERVQUAL model remains one of the most widely used frameworks. SERVQUAL assesses service quality based on five key dimensions, namely tangibility, reliability, responsiveness, assurance and empathy (Marinković et al. 2013; Slamet & Sulistiyowati 2022; Solano-Solano et al. 2023). This model provides valuable insights into the gaps between patient expectations and actual service experiences, guiding health care institutions in targeted quality improvement initiatives.

The urgency of this study is to improve hospital management practices by identifying the factors that most influence patient satisfaction. From a social perspective, improving service quality contributes to better patient experience, increased trust in health care institutions and higher compliance with medical care.

These improvements can lead to positive health outcomes at both individual and community levels. From a scientific standpoint, this study aims to fill the existing gaps in health care quality research, particularly concerning inpatient services in resource-constrained settings. While previous studies have extensively examined service quality in outpatient and private health care facilities, limited research has been conducted on public hospitals in developing countries. This study’s focus on Batara Siang Hospital, a regional health care provider in Indonesia, offers a unique opportunity to understand service quality challenges in such settings and develop evidence-based recommendations for improvement.

The SERVQUAL model serves as the conceptual framework for this study, offering a structured approach to evaluating service quality from a patient-centred perspective. The five dimensions of SERVQUAL, namely tangibility, reliability, responsiveness, assurance and empathy, provide a comprehensive understanding of hospital service performance and areas for enhancement. Tangibility refers to the physical aspects of hospital services, including cleanliness, facilities and medical equipment. Reliability assesses the consistency and accuracy of health care service delivery. Responsiveness measures the willingness and promptness of hospital staff in assisting patients. Assurance reflects the competence, courtesy and trustworthiness of health care professionals. Empathy evaluates the degree of personalised care and understanding shown to patients. This framework allows for the identification of key service dimensions that impact patient satisfaction, enabling health care managers to implement targeted interventions aimed at improving overall hospital service quality.

This study aims to analyse patient satisfaction with inpatient services at Batara Siang Hospital using the SERVQUAL model. Specifically, the study seeks to assess the perceived quality of inpatient services across the five SERVQUAL dimensions, determine the relative influence of each SERVQUAL dimension on overall patient satisfaction, identify areas for improvement in hospital service quality based on patient feedback and provide evidence-based recommendations for health care administrators to enhance patient satisfaction and hospital performance.

## Research methods and design

### Study design

This study used a cross-sectional design with quantitative methods to comprehensively assess patient satisfaction with inpatient services at Batara Siang Hospital in Pangkep, Indonesia. The study was conducted from January 2023 to October 2023, beginning with a pre-test in January, followed by full-scale data collection over a period of 10 months to ensure a comprehensive and representative timeframe of patient experiences within the hospital setting. The SERVQUAL framework, which evaluates the service quality across the five dimensions, namely tangibility, reliability, responsiveness, assurance and empathy, guided the assessment (Jonkisz, Karniej & Krasowska 2021). By integrating quantitative and qualitative approaches, the study achieved both breadth and depth, allowing numerical insights to be enhanced by patients’ lived experiences (Creswell & Clark 2018).

### Setting

The research was conducted in the inpatient wards of Batara Siang Hospital, a public regional health care facility serving a diverse population in South Sulawesi. This hospital reflects typical challenges in Indonesia’s public health care system, such as limited infrastructure and staffing. The inpatient setting provided the ideal environment to capture prolonged patient–provider interactions, which are critical to shaping patient satisfaction (Li, Cui & Feng 2024).

### Study population and sampling strategy

For the quantitative component, the study recruited 267 inpatients aged 25–50 years using purposive sampling. This age group represented the most active demographic based on hospital utilisation data (Yehia et al. 2010). The final sample size of 267 respondents was determined based on hospital admission records during the study timeframe, aiming for adequate coverage across various inpatient wards. Although probabilistic sampling was not feasible, efforts were made to ensure diversity by including patients from different service units and backgrounds. All participants were surveyed upon discharge to ensure they had completed their inpatient care experience. Inclusion criteria required a minimum 24-h hospital stay, cognitive and physical ability to participate and informed consent. Patients in critical care or with cognitive impairments were excluded to preserve data reliability.

For the qualitative component, a subsample of 12 patients was purposively selected from the survey respondents using maximum variation sampling to ensure demographic and service diversity. Participants were drawn from different wards, gender groups and education levels. Sampling continued until thematic saturation was reached, that is, when no new insights were emerging from additional interviews (Leese et al. 2021).

### Data collection instruments and procedures

Quantitative data were collected using a structured SERVQUAL questionnaire consisting of 22 items linked to the five core dimensions. Responses were measured using a 5-point Likert scale ranging from 1 (strongly disagree) to 5 (strongly agree). The instrument was adapted and pilot tested with 30 patients to ensure cultural relevance and clarity. The pilot results yielded a Cronbach’s alpha above 0.80 across all dimensions, indicating strong internal consistency (Utami & Supriadi 2023). Minor revisions to item wording were made based on pilot feedback to improve comprehensibility.

Qualitative data were gathered through semi-structured interviews, guided by open-ended questions aligned with SERVQUAL dimensions. Interviews were conducted face to face in Bahasa Indonesia by trained facilitators in private hospital rooms or offices. Each session lasted 25 min – 30 min and was audio-recorded with participant consent. Transcriptions were later translated into English and validated through back-translation to ensure semantic accuracy.

### Data analysis

Quantitative data were analysed using SPSS version 26 (Mitra 2023). Descriptive statistics summarised participant demographics, and multiple linear regression was conducted to examine the influence of SERVQUAL dimensions on overall satisfaction. The regression model was tested for statistical assumptions, including linearity, independence, multicollinearity and normality. Significance was set at *p* < 0.05.

Qualitative data were analysed thematically following Braun and Clarke’s six-phase approach (2023). Transcripts were coded independently by two researchers, and emerging patterns were reviewed collaboratively. Discrepancies were resolved through consensus. To enhance rigour, the analysis included audit trails, coder triangulation and peer debriefing (Nowell et al. 2017).

Thematic analysis was conducted to identify patterns and select interesting patterns related to the research objectives with the following steps: (1) The three research groups completed reading the interview transcripts, and the initial themes identified from the reading were used to produce a framework as initial material for analysis; (2) In-depth reading and coding of the interview transcript material; (3) The results of the coding and organisation of data will produce several themes; (4) The results of the coding analysis are rechecked by cross-checking the raw data that have been coded; (5) The assessment and interpretation of the selected themes are then discussed comprehensively in the research group to determine whether and how the selected themes represent responses or meanings in the interview transcript data set; (6) The final results yield several themes, namely 2 themes (safety in performing work and safety in receiving support) and 1 subtheme (sense of safety felt when first starting work). The qualitative analysis of these themes is used for quantitative triangulation, providing a deeper context for understanding the gaps in patient satisfaction and priorities as the final analysis results (Braun & Clarke 2023; Nowell et al. 2017; Wall, Svensson & Berg Jansson 2021).

### Methodological rigour and trustworthiness

To enhance methodological credibility, the study incorporated multiple strategies for rigour: (1) Credibility was supported by triangulating data sources and analyst agreement, (2) Transferability was enhanced by including diverse patient profiles and describing the study setting in detail, (3) Dependability was achieved through consistent application of structured procedures and (4) Confirmability was maintained through transparent documentation and external verification (Ahmed 2024). These methodological features provide confidence that the study findings are both reliable and meaningful, adding value for hospital administrators seeking to improve inpatient service quality.

### Ethical considerations

Ethical approval for the study was obtained from the Health Research Ethics Committee of Makassar Health Polytechnic (approval number 1070/M/KEPK-PTKMS/VII/2022). Written informed consent was obtained from all participants before data collection, ensuring voluntary participation and confidentiality. Approval and cooperation were also secured from relevant gatekeepers at Batara Siang Hospital, including hospital administrators and ward coordinators, to facilitate access to participants and ensure compliance with institutional procedures. To maintain anonymity, no personally identifiable information was collected. Participants were informed of their right to withdraw from the study at any time without consequences.

## Results

### Sociodemographic characteristics of respondents

The study included 267 inpatients aged 25–50 years, with a mean age of 34.39 years (s.d. ± 11.86). This age group was selected because it represents the most frequent users of inpatient services at Batara Siang Hospital, typically characterised by active working-age adults with diverse health care needs and service expectations. The majority of respondents were female (55.4%), while males comprised 44.6%. Educational background varied, with 43.4% having completed primary education, 40.1% secondary education and 16.5% higher education. Patients with lower educational levels may have limited health literacy, which can influence both their understanding of care processes and their expectations, potentially leading to lower satisfaction when services are perceived as unclear or insufficiently explained (Hadden et al. 2018). In terms of employment, 51.3% were employed and 48.7% unemployed. Employment status may shape expectations around service efficiency, and communication employed patients, for instance, may prioritise timeliness and clear instructions because of time constraints (Mazzi et al. 2020). The demographic composition suggests a diverse sample whose varying socio-economic characteristics likely influenced their expectations and perceptions of service quality (Table 1).

**TABLE 1:** Sociodemographic characteristics among 267 research participants at Batara Siang General Hospital, Pangkep.

Characteristics	Total				*p*
	Mean	s.d.	*n*	%	
**Age, mean ± s.d (years)**	34.39	± 11.86			0.001
< 20	-	-	30	11.2	
20–29	-	-	75	28.1	
30–39	-	-	76	28.5	
40–49	-	-	62	23.2	
> 50	-	-	24	9.0	
**Sex**					
Male	-	-	119	44.6	0.001
Female	-	-	148	55.4	
**Education level**					
Basic education	-	-	116	43.4	0.001
Moderate education	-	-	107	40.1	
High education	-	-	44	16.5	
**Employment status**					
Employed	-	-	137	51.3	0.001
No	-	-	130	48.7	

s.d., standard deviation.

### SERVQUAL dimensions: Gaps in expectations versus perceptions

The findings demonstrated that all five SERVQUAL dimensions significantly influenced patient satisfaction. The highest impact was recorded for the empathy dimension (β = 0.629, *p* < 0.001), suggesting that patients value personalised and compassionate care. Assurance (β = 0.502, *p* < 0.001) ranked second, indicating that patients place a high level of importance on the competence and credibility of health care providers. Responsiveness (β = 0.479, *p* < 0.001) followed, showing that timely and efficient service delivery is crucial in shaping patient perceptions of quality. Reliability (β = 0.315, *p* = 0.01) and tangibility (β = 0.305, *p* = 0.04) also contributed significantly to satisfaction, emphasising the role of service consistency and the physical hospital environment in overall patient experiences (Table 2).

**TABLE 2:** Measurements or observations related to patient satisfaction in health care settings.

Dimensions	Items	Answer of belief							Answer of belief							Gap
		STS	TS	N	S	SS	∑	X	STS	TS	N	S	SS	∑	Y	
Reliability	1	0	102	87	68	10	787	2.95	0	0	5	145	117	1180	4.42	
	2	1	111	61	76	18	800	3.00	0	0	4	134	129	1193	4.47	
	3	0	2	97	155	13	980	3.67	0	0	6	149	112	1174	4.40	
	4	0	86	78	86	17	835	3.13	0	0	9	600	570	1179	4.42	
	Total average: 856 or grand mean 3.20								Total average: 1.182 or grand mean 4.43							−1.23
Tangibility	4	1	13	152	82	19	906	3.39	0	0	6	147	114	1176	4.40	
	5	0	25	139	86	17	896	3.36	0	0	5	142	120	1183	4.43	
	6	0	27	110	114	16	920	3.45	0	0	4	140	123	1187	4.45	
	7	0	31	107	114	15	914	3.42	0	0	5	134	128	1191	4.46	
	Total average: 909 or grand mean 3.40								Total average: 1.184 or grand mean 4.44							−1.04
Responsibility	8	1	119	100	28	19	746	2.79	0	0	6	132	129	1191	4.46	
	9	1	26	124	102	14	903	3.38	0	0	5	131	131	1194	4.47	
	10	1	121	110	23	12	725	2.72	0	0	4	137	126	1190	4.46	
	11	0	127	98	23	19	735	2.75	0	0	5	136	126	1189	4.45	
	Total average: 777 or grand mean 2.91								Total average: 1.191 or grand mean 4.46							−1.55
Assurance	12	1	119	115	22	10	722	2.70	0	-	7	138	122	1183	4.43	
	13	2	109	122	21	13	735	2.75	0	0	6	142	119	1181	4.42	
	14	1	129	102	20	15	720	2.70	0	0	8	134	125	1185	4.44	
	15	0	102	126	27	12	750	2.81	0	0	4	136	127	1191	4.46	
	Total average: 732 or grand mean 2.74								Total average: 1.185 or grand mean 4.44							−1.70
Empathy	16	1	127	102	25	12	721	2.70	0	0	5	132	130	1061	3.97	
	17	2	128	100	22	15	721	2.70	0	0	4	137	126	1190	4.46	
	18	1	123	102	26	15	732	2.74	0	0	3	133	131	1196	4.48	
	19	0	94	132	27	14	762	2.85	0	0	9	129	129	1188	4.45	
	Total average: 734 or grand mean 2.75								Total average: 1.159 or grand mean 4.34							−1.59
	**Total average: 802 or grand mean 3.00**								**Total average: 1.159 or grand mean 4.34**							**−1.42**

### Multivariate analysis of SERVQUAL dimensions

Table 2 summarises the comparison between patients’ expectations and perceptions across the five SERVQUAL dimensions. All dimensions showed a negative satisfaction gap, indicating that expectations exceeded the actual service experienced.

Tangibility had the smallest gap (–1.04), suggesting that patients were relatively more satisfied with the hospital’s physical infrastructure and cleanliness. In contrast, assurance showed the widest gap (–1.70), followed by empathy (–1.59) and responsiveness (–1.55), highlighting critical areas of concern regarding communication, personal attention and professional confidence.

### Demographic-based variations in satisfaction

**FIGURE 1:**
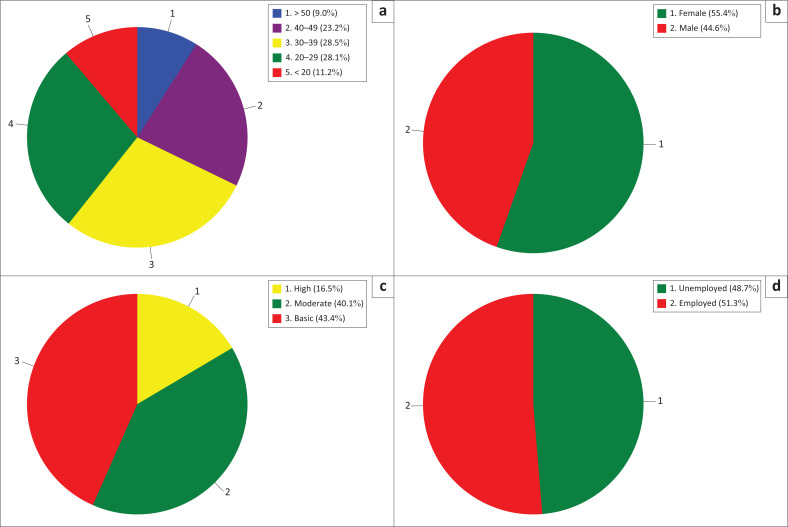
Distribution of patient satisfaction in each SERVQUAL dimension. (a) Age, (b) gender, (c) education level and (d) employment status.

Figure 1 presents satisfaction distribution across age, gender, education and employment status. It shows that younger patients experienced higher dissatisfaction with empathy and responsiveness. Participants with higher education levels were more critical of reliability and assurance, while female patients reported lower satisfaction in tangibility and assurance dimensions.

### Graphical visualisation of satisfaction gaps

**FIGURE 2:**
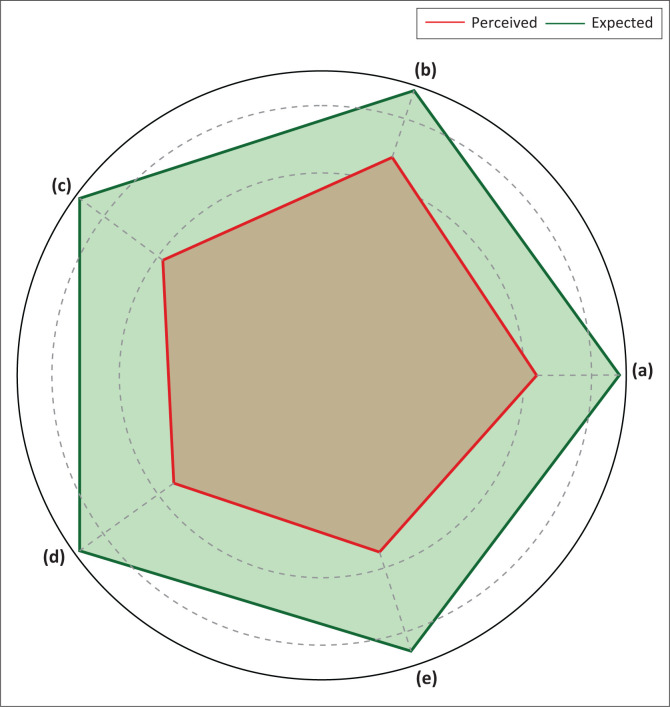
Pattern of patient satisfaction in various dimensions of SERVQUAL. The green line represents patient expectations, while the red line indicates perceived satisfaction. (a) Tangibility, (b) reliability, (c) responsiveness, (d) assurance and (e) empathy.

Figure 2 graphically displays the patterns of expectation versus perception across all five dimensions. The green line represents patient expectations, while the red line indicates perceived service quality. The steepest differences are found in the dimensions of assurance, empathy and responsiveness. The reliability dimension shows that patients feel that the service is still not consistent or reliable enough, with an actual satisfaction score of 3.20 compared to expectations of 4.43 (Figure 2a). Tangibility is the dimension with the highest satisfaction among others (3.40), but it is still lower than patients’ expectations of 4.44, indicating that physical aspects such as hospital facilities and cleanliness still need to be improved (Figure 2b). Responsiveness shows a sizable gap, with satisfaction of 2.91 compared to expectations of 4.46, indicating that patients feel health workers are not quick enough to provide services (Figure 2c). Assurance is the dimension with the lowest satisfaction (2.74), far below expectations (4.44), indicating that patients lack confidence in the competence of medical personnel in providing care (Figure 2d). Meanwhile, the empathy dimension also showed considerable dissatisfaction, with a score of 2.75 compared to the expectation of 4.34, meaning that patients felt less personally cared for in the health services they received (Figure 2e).

## Discussion

This study evaluated inpatient satisfaction at Batara Siang Hospital using a mixed-method SERVQUAL approach, revealing persistent negative satisfaction gaps across all five dimensions, particularly in assurance, empathy and responsiveness.

Patient expectations were shaped by several demographic factors. The predominance of individuals in the productive age group (25–50 years) likely contributed to higher expectations for prompt, efficient and goal-oriented services. The findings of this study suggest that participant characteristics, particularly age and education, significantly influenced satisfaction in key service dimensions. Younger patients, especially those aged 25–39 years with higher education levels, consistently reported larger negative satisfaction gaps in assurance, empathy and responsiveness (Johnson et al. 2019; Lin et al. 2021; Nichols et al. 2021). This trend supports the expectancy–disconfirmation framework, which posits that more informed or empowered individuals are more likely to experience dissatisfaction when services fall short of perceived standards (Filtenborg, Gaardboe & Sigsgaard-Rasmussen 2017; Zhang et al. 2022). Similar patterns were reported in a study from Ethiopia, where patients with higher education levels showed lower satisfaction, likely because of greater expectations and health literacy (Wudu 2021). However, contrasting results from Rwanda found that less-educated patients were more dissatisfied, likely because of poor communication and inadequate service orientation (Sebera, Hagenimana & Twagirumukiza 2024). These findings highlight how the relationship between education and satisfaction may vary based on cultural and institutional factors but consistently point to the role of expectations in shaping service evaluations.

Beyond education, patient satisfaction was also shaped by prior hospital experience, frequency of visits and socio-economic factors. Respondents who had been hospitalised multiple times were more likely to detect inconsistencies in service, especially during weekends or staff transitions, which contributed to dissatisfaction with reliability. These insights are consistent with findings from Saudi Arabia, where repeat visits were associated with higher scrutiny and lower satisfaction when services lacked consistency (Elias et al. 2022). On the contrary, a 2023 systematic review from Ethiopia noted that demographic variables such as education or residence were not always significant predictors of satisfaction but emphasised the importance of respectful communication, privacy and timely information (Mulugeta et al. 2019). Our results reinforce that while demographic characteristics influence the lens through which service is experienced, core relational factors such as empathy and responsiveness remain universal in determining how patients evaluate their care. This supports the need for hospitals to align both individual-level expectations and system-wide relational practices to improve satisfaction (Lv et al. 2025).

Female patients, who constituted the majority, may have placed greater emphasis on communication, empathy and personal attention. Those with lower levels of education may have faced challenges in fully understanding clinical procedures and their rights, leading to expectations for clearer explanations and more guided care. The findings indicate that female patients, who made up the majority of respondents, tended to place greater emphasis on communication, empathy and personal attention. This aligns with previous studies showing that women are more attuned to relational dynamics in care settings and are more likely to report dissatisfaction when emotional needs are unmet (Álvarez-Díaz 2020; Suhonen et al. 2018; Teunissen, Rotink & Lagro-Janssen 2016). Recent research by Cantalino et al. (2021) in Brazil and Liu et al. (2023) in China revealed similar patterns, noting that female patients scored lower in perceived empathy and assurance despite receiving comparable technical care. Moreover, this heightened sensitivity may stem from sociocultural roles where women often assume caregiver responsibilities, giving them higher relational expectations. However, a contrasting study by Alemu et al. (2024) in Ethiopia found no significant gender difference in satisfaction scores, suggesting that contextual variables such as staff–patient ratios and cultural norms may mediate gendered perceptions of care. Thus, while our findings support global evidence on gender-related expectations, they also reveal that these dynamics are not uniform across settings and demand context-specific interpretation. In parallel, patients with lower levels of education were more likely to report a need for clearer explanations and guided care, echoing concerns around health literacy and provider communication.

This is supported by studies across Southeast Asia and sub-Saharan Africa, which found that patients with limited formal education often rely heavily on the relational quality of health care interactions to compensate for their difficulty in interpreting medical information (Forray et al. 2024; Gwaza et al. 2024). However, this relationship is nuanced. While some literature suggests that less-educated patients are more tolerant of poor service because of limited expectations, recent findings by Kwame and Petrucka (2021), showed that even patients with low education levels express dissatisfaction when communication is dismissive or rushed. These contradictory trends emphasise that education level may not solely dictate expectations but rather influences how patients interpret the intent and delivery of care. This reinforces the need for service providers to adapt communication styles, not just content, based on each patient’s ability to understand and emotionally process information.

Tangibility had the smallest gap (–1.04), suggesting general patient satisfaction with the hospital’s physical infrastructure. Comparable results were observed in Ebonyi State, Nigeria (Umoke et al. 2020), where patients expressed relative satisfaction with physical infrastructure but noted room for improvement (Singh & Sidhu 2023). Similar findings have been reported by Meng and Chen (2025), who noted that while infrastructure matters, it plays a lesser role in shaping patient loyalty compared to interpersonal service quality. Reliability, although statistically significant, had a moderate effect indicating that consistency alone does not ensure patient satisfaction.

The most critical gaps were found in assurance, empathy and responsiveness. These are consistent with global studies in both developed and developing health care systems. For instance, a study by Liu et al. (2024) in China and a mixed-method study by Kamgba (2023) in Nigeria also highlighted patient dissatisfaction stemming from a lack of empathy, unclear communication and delayed service. Our regression model similarly confirmed empathy (β = 0.629), assurance (β = 0.502) and responsiveness (β = 0.479) as the most influential factors.

This study also revealed how demographic factors shape expectations. Younger patients and those with higher education levels expected quicker service and clearer communication, aligning with findings by Charles, Sivayokan and Kumanan (2024), Cappella and Street (2024) and Nembhard et al. (2023), who emphasised the importance of adjusting communication strategies based on patient profiles. The importance of assurance and responsiveness also mirrors the findings of Salsabila et al. (2023), who highlighted that patients often assess quality based on staff competency and the speed of care delivery.

The integration of qualitative interviews confirmed and contextualised these findings. Patients expressed frustration with slow response times and a perceived lack of attention, which supports prior research advocating for a mixed-method approach to hospital service evaluation (Howick et al. 2024a; Xiong et al. 2025). This triangulated perspective strengthens the interpretive power of the SERVQUAL dimensions.

Importantly, although tangibility received the highest satisfaction mean score, its regression coefficient was the lowest, suggesting that patients prioritise relational care over physical improvements. This matches the findings by Abideen, Obamiro and Tijani (2024) and Araujo, Siqueira and Malik (2020), who concluded that patient-centred behaviours contribute more significantly to satisfaction than environmental aesthetics.

While the study’s SERVQUAL-based structure and statistical strength are notable, its limitations include a single-hospital focus and a cross-sectional design. Future studies should include more hospitals, varied settings and longitudinal approaches. Moreover, qualitative interviews should be expanded to better understand patient narratives and emotional experiences.

The findings validate the SERVQUAL model’s utility and highlight that in hospital care, how patients are treated matters more than how facilities appear. Empathy, responsiveness and assurance, not just cleanliness or equipment, determine whether patients feel truly cared for. Addressing these dimensions is essential for any hospital aiming to improve its quality of care.

### Implications for healthcare management

The study provides critical insights for hospital administrators and policymakers, particularly in public health care settings. The findings clearly indicate that empathy, assurance and responsiveness were the most influential dimensions driving patient satisfaction. Therefore, these aspects should be prioritised in hospital quality improvement strategies. For instance, the significant role of empathy suggests a need to invest in staff training programmes that enhance patient-centred communication, active listening and compassionate care (Howick, De Zulueta & Gray 2024b; Kang et al. 2022).

This priority aligns with the Lean Six Sigma framework, an integrated improvement approach that improves service quality by reducing variation, defects and costs. In health care, the Lean Six Sigma framework accelerates access to health care services with no waiting times, while reducing defects means fewer complications. Increasing speed and reducing defects both result in lower costs. Therefore, Lean Six Sigma is an excellent tool for addressing today’s health care challenges (McCollin et al. 2011). Hospitals can adopt structured training modules and implement leadership practices that foster an empathic organisational culture. In addition, the focus on assurance and responsiveness supports streamlining operational processes such as reducing waiting times, improving staff competency and ensuring prompt responses to patient needs (Al Rukhami et al. 2024). These elements were reflected in patient dissatisfaction scores found in our SERVQUAL analysis, particularly in responsiveness and assurance dimensions.

To measure the effectiveness of such interventions, hospitals should strengthen their use of key performance indicators (KPIs). Relevant KPIs in this context may include average patient waiting time, time to first response by staff, patient complaint resolution time, patient-reported satisfaction with staff communication and staff-to-patient ratios (Khan et al. 2023). These indicators provide tangible metrics for tracking improvements in both operational efficiency and interpersonal service quality. Lean Six Sigma is a programme that can help health care providers achieve these seemingly conflicting goals. Lean Six Sigma is a programme for improving the quality of services, particularly hospital health care services. Hospitals today face enormous challenges. Patients demand continuous improvement in the quality of care. Health insurance companies demand the lowest possible prices. The Lean Six Sigma framework accelerates health care services with quick access and no waiting time, while reducing defects means reducing complications. Increasing speed and reducing defects both result in lower costs. Therefore, Lean Six Sigma is an excellent tool for addressing today’s health care challenges. Furthermore, integrating methodologies such as Lean and Six Sigma can help streamline hospital workflows and reduce delays, thereby enhancing responsiveness and overall patient experience (ALObaid et al. 2024; Alzain et al. 2024; Chowdhury, Chowdhury & Abdullah 2024). Investments in infrastructure, while still important, should be aligned with service delivery KPIs to ensure a balanced, patient-centred approach (Sanchez Leitner et al. 2025).

In terms of implications, this study offers three actionable recommendations for hospital managers: (1) prioritise empathy and communication in staff training, (2) reduce response times through workflow optimisation and use of KPIs like patient wait time and staff responsiveness and (3) implement continuous quality monitoring via tools such as Lean or Six Sigma (Sanchez Leitner et al. 2025; Thakur, Akerele & Randell 2023). These strategies are in line with global quality improvement frameworks and can enhance both service delivery and patient trust.

### Strengths of the study

The SERVQUAL model provided a comprehensive and structured framework to evaluate inpatient service quality at Batara Siang Hospital by examining five critical dimensions, namely tangibility, reliability, responsiveness, assurance and empathy. Tangibility allowed the study to capture the physical infrastructure, cleanliness and medical facilities, which patients generally perceived positively. Reliability ensured the assessment of consistent service delivery and accuracy of care processes. Responsiveness highlighted the timeliness of health care staff in meeting patient needs, while assurance focused on competence and credibility, providing insights into patients’ trust in the hospital workforce. Most importantly, empathy emphasised the relational and humanistic aspects of care, which emerged as the strongest predictor of satisfaction (Narendra & Yadav 2024; Sorathiya & Patel 2024; Utkirov 2024). By combining these dimensions within a mixed-methods design, the study not only quantified satisfaction levels but also contextualised patient experiences, offering hospital administrators practical and evidence-based recommendations for targeted quality improvements.

### Limitations of the study

Despite its strengths, the SERVQUAL model in this study also presents some limitations when applied across the five dimensions. While tangibility was assessed, the model may not fully capture cultural preferences or context-specific expectations regarding hospital facilities. Reliability and responsiveness, although significant, were constrained by the cross-sectional design, which could not account for temporal variations in service delivery, such as staff rotation or peak patient loads. Assurance, while valuable in evaluating provider competence, is influenced by broader systemic issues like staffing shortages and resource constraints that the model cannot fully address. Empathy, though the strongest factor, remains subjective and may vary depending on patients’ personal, educational and socio-cultural backgrounds. Furthermore, the study’s single-hospital setting limits the generalisability of findings across different health care institutions. Thus, while SERVQUAL effectively identifies satisfaction gaps, its scope should be complemented with longitudinal and multi-centre approaches to capture more dynamic and context-sensitive insights.

### Future research directions

To address these limitations, future studies should consider employing longitudinal designs to capture trends in patient satisfaction over time. Expanding the scope to include multiple hospitals and outpatient services would provide a more comprehensive understanding of service quality in diverse health care settings. Additionally, qualitative research methods such as in-depth interviews could complement quantitative findings by offering deeper insights into patient expectations and experiences.

## Conclusion

This study aimed to assess inpatient service quality at Batara Siang Hospital using the SERVQUAL model through a mixed-methods approach. The quantitative findings revealed consistent negative gaps across all service dimensions, with assurance, empathy and responsiveness emerging as the most critical factors influencing patient satisfaction. Qualitative data added contextual depth, uncovering real patient experiences behind the statistical gaps, particularly frustration with delayed responses and impersonal interactions. These findings confirm that relational care elements significantly outweigh environmental factors in shaping satisfaction. The mixed-method design, as discussed, increased the study’s methodological rigour and produced findings consistent with global literature on hospital service quality.

Looking ahead, future research should explore multi-centre studies across varied hospital types to enhance generalisability and allow comparative benchmarking. Longitudinal studies are also recommended to track the impact of service improvement initiatives over time. Additionally, expanding the qualitative dimension – particularly through patient journey mapping or narrative inquiry – could further illuminate emotional and behavioural aspects of patient experiences. Embedding SERVQUAL within hospital quality management systems and aligning it with frameworks like Lean or Six Sigma may support more responsive, human-centred health care delivery in Indonesia and beyond.
